# Prevalence and adverse effects of sport-related nutritional supplements (sport drinks, bars, and gels) in the military before and during the COVID-19 pandemic: the US Military Dietary Supplement Use Study

**DOI:** 10.1080/15502783.2023.2277246

**Published:** 2023-11-10

**Authors:** Joseph J Knapik, Daniel W Trone, Ryan A Steelman, Emily K Farina, Harris R Lieberman

**Affiliations:** a US Army Research Institute of Environmental Medicine, Military Nutrition Division, Natick, MA, USA; b Military Population Health Directorate, Naval Health Research Center, San Diego, CA, USA; c Clinical Public Health & Epidemiology, Defense Centers for Public Health - Aberdeen, Aberdeen Proving Ground, MD, USA

**Keywords:** Nutrition, gender, age, body mass index, physical activity, dietary supplements

## Abstract

**Background:**

Sport-related nutritional supplements (SRNS) include sport drinks, sport bars, and sport gels. This investigation examined temporal patterns in SRNS use and adverse effects (AEs) reported by a single cohort of United States active-duty service members (SMs) surveyed before and during the coronavirus disease 2019 (COVID-19) pandemic.

**Methods:**

A stratified random sample (*n* = 22,858) of SMs (Air Force, Army, Navy, and Marine Corps) who completed a questionnaire on their SRNS use and AE experiences and were still on active duty were asked to complete the identical questionnaire on a second occasion. Twenty-five percent of successfully contacted SMs completed both questionnaires (*n* = 5,778) and were included in this investigation. The average ± standard deviation time between questionnaires was 1.3 ± 0.2 years.

**Results:**

Prevalence of reported SRNS use ≥1 time/week in the baseline (BL) and follow-up (FU) phases were as follows: any SRNS: BL = 46%, FU = 41%; sport drinks: BL = 31%, FU = 28%; sport bars: BL = 30%, FU = 24%; sport gels: BL = 4%, FU = 4%. Reported weekly aerobic and resistance training durations were reduced in the FU period (8% and 26%, respectively). The proportion of SMs reporting SRNS use in both study phases was as follows: any SRNS = 62%, sport drinks = 54%, sport bars = 50%, sport gels = 35%. Prevalence of reported AEs in the BL and FU phases were as follows: any SRNS: BL = 1.9%, FU = 1.9%; sport drinks: BL = 1.0%, FU = 1.3%; sport bars: BL = 1.7%, FU = 1.4%; sport gels: BL = 3.3%, FU = 2.5%. The proportion of SMs reporting AEs in both phases was as follows: any SRNS = 14%, sport drinks = 11%, sport bars = 17%, sport gels = 0%.

**Conclusions:**

Overall SRNS use prevalence decreased slightly in the FU period, possibly because of reduced physical training related to military restrictions imposed during the emergence of COVID-19 between surveys. A large proportion of SMs reported changing their use patterns in the FU with some discontinuing use and others initiating use. The AE incidence was similarly low in the BL and FU phases, and few SMs reported AEs in both phases suggesting AEs were transitory. AE reporting for SRNSs was much lower than previously found for dietary supplements, possibly because of greater government regulatory control over SRNSs.

## 1. Background

Sport-related nutritional supplements (SRNS) include sport drinks, sport bars, and sport gels. These substances are typically used before, during, or after exercise to provide hydration nutrients, and other substances. Sport drinks are typically carbohydrate–electrolyte solutions, while sport bars are generally composed of protein and carbohydrate macronutrients with vitamins and minerals. Sport gels are semi-solid (viscous) substances comprised primarily of quick-digesting carbohydrate sources [1,2]. In addition to the ingredients mentioned above, many SRNSs contain caffeine and various types of flavoring with some containing dietary fiber. The use of SRNS is common among athletes and military personnel with 25% to 35% of the athletes [3] and 25% to 50% of the United States (US) military personnel [4–7] reporting use of these substances.

SRNS consumption is increasing in the US and globally. Sport drink sales in the US amounted to $6.5 billion in 2021 and is projected to reach $9.4 billion by 2029 with an estimated compound annual growth rate of 4.8% [8]. The global market for sport bars was estimated at $4.5 billion in 2021 and expected to increase to $7.1 billion by 2029 with a projected compound annual growth rate of 6.1% [9]. The global energy gel market is considerably smaller than the market for sport drinks and sport bars amounting to $637 million in 2022, but the compound annual growth rate is expected to exceed sport drinks and bars and reach 8.0% with a market value of $1.3 billion by 2032 [10]. These expanding markets may be associated with an increased emphasis on exercise and fitness, as well as increased demand for easily consumed convenience foods [9,10].

There have been studies examining the prevalence of SRNS use in separate samples of US Army [4], Air Force [5], Navy/Marine Corps [6], and Coast Guard [11] personnel, and we recently reported the prevalence of SRNSs and adverse effects (AEs) experienced by a stratified random sample of SMs from all service branches [7]. The purpose of the current investigation was to expand on this research [7] by examining longitudinal trends in SRNS use and reported AEs in a large cohort of US active-duty service members (SMs) followed over time. Tracking the same cohort over time allowed an examination of changes in patterns of SRNS use and AE reporting, for example by providing information on the proportion of individuals who continue SRNS use, discontinue use, and become new users in the period. Coincidentally, the global coronavirus disease 2019 (COVID-19) pandemic emerged between the two administrations of the survey.

## 2. Methods

This investigation used a survey that was completed twice by a subset of a stratified random sample of US active-duty military SMs and was part of a larger study examining dietary supplement use, SRNS use, and AEs [12–14]. The Naval Health Research Center Institutional Review Board approved the investigation and participants signed an informed consent document. Investigators adhered to policies and procedures for protection of human subjects as prescribed by Department of Defense Instruction 3216.01, and the research was conducted in adherence to provisions of 32 Code of Federal Regulations, Part 219.

### 2.1. Sampling frame and solicitation procedures

There were two phases of this study: baseline (BL) and follow-up (FU). Details of the sampling frame, solicitation of SMs, subject recruitment flow chart, statistical power, and response bias in the BL and FU phases have been previously reported [12,13]. Briefly, investigators requested from the Defense Manpower Data Center a random sample of 200,000 SMs stratified by sex (88% male and 12% female) and branch of service (Army 36%, Air Force 24%, Marine Corps 15%, and Navy 25%). Recruitment of the randomly selected SMs into the BL phase involved a maximum of 12 sequential contacts. These included an introductory postal letter, a follow-up e-mail message after 10 days, a postcard 3 weeks later, and up to seven e-mails and three post card reminders evenly distributed across the time the survey was open. After this, contact with the SM ended. All postal and online contacts stated that at any time the SM could decline participation and be removed from the contact list. Recruitment into the BL phase began in December 2018 and ended in August 2019.

As part of the BL informed consent, potential participants were informed there would be an FU phase that would involve the same procedures. Prior to the FU phase, Defense Manpower Data Center identified SMs no longer on active duty so they would not receive an FU request. Other SMs who volunteered for the BL phase and were still on active duty were asked to participate in the FU phase in a letter sent about 8 months after the BL phase closed. Solicitation procedures for the FU phase were the same as in the BL phase, with 12 sequential contacts. Recruitment into the FU phase began in April 2020 and ended in December 2020. The FU phase was conducted shortly after the declarations by the World Health Organization of the global COVID-19 pandemic [15].

### 2.2. Survey description

**Figure 1.**
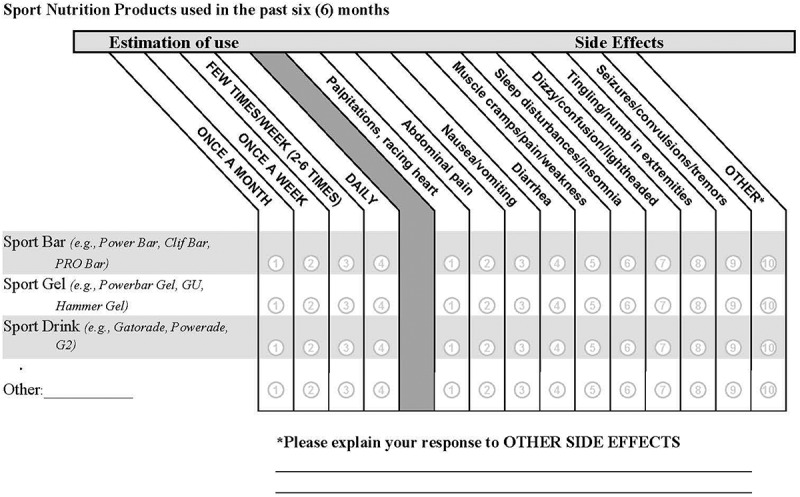
Questionnaire items on frequency of sports-related nutritional supplement use and adverse effects experienced.

The identical survey was used in the BL and FU phases and was based on previous questionnaires of this type [16]. It was completed by participants online. The survey was designed to (1) describe participants’ physical characteristics and lifestyle factors, (2) obtain frequencies of SRNS use by category, and (3) ascertain AEs associated with use of SRNSs. To characterize participants, there were questions on demographics (gender, age, height, weight), and lifestyle factors (weekly duration of aerobic training, weekly duration of resistance training, alcohol consumption). There were four SRNS questions that asked SMs about the frequency of use and AEs associated with (1) sport drinks, (2) sport bars, (3) sport gels, and (4) other. Commercial examples were provided for each SRNS category. The “other” option was provided in case the participant could not categorize the SRNS and space was provided to enter the supplement name or type. For each SRNS, SMs were asked to estimate how frequently the supplement was consumed in the past 6 months. A list of AEs was located alongside each SRNS. The AE list included symptoms related to cardiovascular, gastrointestinal, muscular, sleep disturbance, and neurological symptoms. Figure 1 shows the SRNS questions.

### 2.3. Data analysis

All statistical analyses were conducted using the Statistical Package for the Social Sciences (Version 27, 2019, SPSS Inc.). Body mass index (BMI) was computed from the survey responses as weight/height^2^ (kg/m^2^). Weekly duration of aerobic and resistance training (min/week) was calculated by multiplying reported weekly exercise frequency (sessions/week) by the reported duration of training (min/session). Alcohol consumption was quantified under the National Institute of Health assumption that a “standard drink” contained 17.74 ml of alcohol [17]. “Standard drinks” included 12 oz of regular beer or fermented fruit drink (5% alcohol), 8.5 oz of higher alcohol beer (7% alcohol), 5 oz of wine (12% alcohol), 6.25 oz of fortified wine (15% alcohol), and 1.5 oz of liquor (40% alcohol). If an SM listed “other” for a SRNS, these were individually examined and placed into the proper category (Table 1).

**Table 1.** Comparison of Demographic and lifestyle factors in baseline and follow-up phases.

Gender	Variable	*N*	Baseline Mean ± SD	Follow-up Mean ± SD	Difference (%)^a^	Paired *t*-Test *p*-Value
Men & Women	Age (years)	5,770	35.0 ± 8.0	36.3 ± 8.0	3.7	<0.01
	Height (cm)	5,668	176.7 ± 8.8	176.7 ± 8.9	0.0	0.85
	Weight (kg)	5,695	83.3 ± 13.4	84.0 ± 13.7	0.8	<0.01
	BMI (kg/m^2^)	5,612	26.6 ± 3.3	26.8 ± 3.5	0.8	<0.01
	Aerobic exercise (min/week)	5,770	227 ± 230	210 ± 214	−7.5	<0.01
	Resistance exercise (min/wk)	5,770	198 ± 243	147 ± 214	−25.8	<0.01
	Alcohol consumption (ml/wk)	5,775	59 ± 102	70 ± 118	18.6	<0.01
Men	Age (years)	4,968	35.1 ± 8.0	36.4 ± 8.0	3.7	<0.01
	Height (cm)	4,870	178.6 ± 7.5	178.6 ± 7.5	0.0	0.42
	Weight (kg)	4,903	85.6 ± 12.1	86.3 ± 2.5	0.8	<0.01
	BMI (kg/m^2^)	4,822	26.8 ± 3.3	27.0 ± 3.4	0.7	<0.01
	Aerobic exercise (min/wk)	4,964	228 ± 231	210 ± 212	−7.9	<0.01
	Resistance exercise (min/wk)	4,964	206 ± 247	151 ± 217	−26.7	<0.01
	Alcohol consumption (ml/wk)	4,970	64 ± 107	75 ± 125	17.2	<0.01
Women	Age (years)	802	34.2 ± 8.4	35.5 ± 8.4	3.8	<0.01
	Height (cm)	798	165.0 ± 7.0	164.8 ± 7.1	0.1	0.10
	Weight (kg)	792	68.6 ± 11.1	69.7 ± 11.7	1.6	<0.01
	BMI (kg/m^2^)	790	25.2 ± 3.5	25.6 ± 3.8	1.6	<0.01
	Aerobic exercise (min/wk)	806	224 ± 225	211 ± 224	−5.8	0.14
	Resistance exercise (min/wk)	806	151 ± 220	123 ± 192	−18.5	<0.01
	Alcohol consumption (ml/wk)	805	27 ± 48	42 ± 62	55.6	<0.01

Abbreviations: BMI,body mass index; SD,standard deviation.

^a^Calculated as follows: (follow-up − baseline)/baseline × 100%.

Descriptive statistics (means and standard deviations) were calculated for the physical characteristics and lifestyle factors, and these were compared in the BL and FU phases with a paired *t*-test. For SMs reporting SRNS use ≥1 time/week, SRNS use and AE prevalence (%) were calculated with their 95% confidence interval (95% CI) or standard error for each SRNS category (Table 1) in the FU and BL phases. Changes in use prevalence and AE reporting between the two phases were calculated. For SRNS use prevalence, two by two tables were constructed to examine continued use, continued nonuse, and changes in use in each SRNS category (i.e. BL use/nonuse by FU use/nonuse). In the same manner, two by two tables were constructed to examine changes in AE reporting in each SRNS category (i.e. BL AE/no AE by FU AE/no AE). The McNemar test for repeated measures [18] was used to compare changes across the BL and FU phases. The odds of SRNS use at FU (≥1 time/week) was examined among those reporting and not reporting BL AEs and the chi-square statistic was used to compare these groups. Use prevalence of SRNS ≥1 time/week were also calculated for each military service (Air Force, Army, Marine Corps, and Navy) and these were compared using the chi-square statistic.

## 3. Results

From the initial sample frame of 200,000 SMs, 73% (*n* = 146,365) were successfully contacted (i.e. no postal mail returned as undeliverable) at BL and of these 26,681 (18.2%) signed the informed consent and completed the BL questionnaire. Of the 26,681 BL responders, 22,858 (86%) were still on active duty at the start of the FU phase and were successfully contacted at least once during FU phase. Of these, 5,778 completed the FU questionnaire for an FU response rate of 25.3% (5,778/22,858). The average ± SD follow-up time (time from BL to FU questionnaire completions) was 15.8 ± 2.0 months with a range of 9.9–22.8 months.

### 3.1. Changes in demographics and lifestyle factors

Table 1 presents descriptive statistics for the demographics and lifestyle factors in the BL and FU phases and compares changes in the two phases. For both men and women, age, body weight, BMI, and alcohol consumption increased in the FU phase. The amount of aerobic and resistance training decreased in the FU phase.

### 3.2. Changes in prevalence and patterns of use by SRNS category

Table 2 provides prevalence of SRNS use in the BL and FU phases. SMs slightly reduced their use of SRNS in the FU phase compared to the BL phase. Sport bars had the largest change and sport gels the smallest.

**Table 2.** Prevalence of sport-related nutritional supplement use in baseline and follow-up phases.

SRNS Category	Baseline		Follow-up		Prevalence Differences (%)^a^	Prevalence Ratio (Follow-up/Baseline)
	*N*	Prevalence % (95% CI)	*n*	Prevalence % (95% CI)		
Any SRNS	2,673	46.3 (44.4–48.2)	2,350	40.7 (38.7–42.7)	−5.6	0.88
Sport Drinks	1,775	30.7 (28.6–32.8)	1,626	28.1 (25.9–30.3)	−2.6	0.92
Sport Bars	1,725	29.9 (27.7–32.1)	1,386	24.0 (21.8–26.2)	−5.9	0.80
Sport Gels	211	3.7 (1.2–6.2)	204	3.5 (1.0–6.0)	−0.2	0.95
Sport Bars & Gels^b^	1,774	30.7 (28.6–32.8)	1,453	25.1 (22.9–27.3)	−5.6	0.82

Abbreviation: SRNS, sports-related nutritional supplement.

^a^Calculated as follows:(follow-up prevalence − baseline prevalence).

^b^This category was included to allow comparisons with the literature because many military studies combine sport bars and gels in reporting prevalences.

Table 3 shows the changes in prevalence by SRNS categories in the FU phases. Among users at BL, 62% to 35% reported consistent use (i.e. use in both BL and FU phases) while 38% to 65% discontinued use in the FU phase. In descending order, categories with the highest prevalence of discontinued use were sport gels, sport drinks, and sport bars. Among non-users at BL 17% to 2% reported use in the FU period while 84% to 98% reported never using in either phase. In descending order, the categories with the largest proportion of new users in the FU phase were sport drinks, sport bars, and sport gels.

**Table 3.** Changes in prevalence of sport-related nutritional supplement use at follow-up.

SRNS Category	Baseline Users				Baseline Non-Users				McNemar Test *p*-Value
	Users at Baseline Reporting Use at Follow-up (Consistent Users)		Users at Baseline No Longer Reporting Use at Follow-up (Discontinued Use)		Non-Users at Baseline Reporting Use at Follow-up (New Users)		Non-Users at Both Baseline and Follow-up (Never Users)		
	*n*	Prevalence (%)	*n*	Prevalence (%)	*n*	Prevalence (%)	*n*	Prevalence (%)	
Any SRNS	1,660	62.1	1,013	37.9	690	22.2	2,415	77.8	<0.01
Sport Drinks	966	54.4	809	45.6	660	16.5	3,343	83.5	<0.01
Sport Bars	861	49.9	864	50.1	525	13.0	3,528	87.0	<0.01
Sport Gels	74	35.1	137	64.9	130	2.3	5,437	97.7	0.69
Sport Bars & Gels^a^	912	51.4	862	48.6	541	13.5	3,463	86.5	<0.01

Abbreviation: SRNS, sport-related nutritional supplement.

^a^This category was included to allow comparisons with the literature because many military studies combine sport bars and gels in reporting prevalences.

Table 4 shows the prevalence of SRNS use by military service. Marine Corps personnel had the highest use of SRNSs in all categories with much smaller differences among other services.

**Table 4.** Prevalence of sport-related nutritional supplement use in baseline and follow-up phases by military service.

Phase	Sport-Related Nutritional Supplement	Air Force (*n* = 2,295) % (95% CI)	Army (*n* = 1,807) % (95% CI)	Marine Corps (*n* = 574) % (95% CI)	Navy (*n* = 1,102) % (95%CI)	Chi-square *p*-Value
Baseline	Any SRNS	43.9 (41.9–45.9)	45.2 (42.9–47.5)	59.8 (55.8–63.8)	46.0 (43.1–48.9)	<0.01
	Sport Drinks	28.1 (26.3–29.9)	30.6 (28.5–32.7)	42.9 (38.9–46.9)	29.9 (27.2–32.6)	<0.01
	Sport Bars	27.1 (25.9–29.5)	28.2 (26.1–30.3)	40.6 (36.6–44.6)	31.4 (28.7–34.1)	<0.01
	Sport Gels	3.1 (2.4–3.8)	3.9 (3.0–4.8)	4.9 (3.1–6.7)	3.9 (2.8–5.0)	0.15
	Sport Bars/Gels^a^	28.5 (26.7–30.3)	29.2 (27.1–31.3)	40.9 (36.9–44.9)	32.5 (29.7–35.3)	<0.01
Follow-up	Any SRNS	37.1 (35.1–39.1)	39.9 (37.6–42.2)	53.1 (49.0–57.2)	42.8 (39.9–45.7)	<0.01
	Sport Drinks	25.2 (23.4–27.0)	28.7 (26.6–30.8)	38.3 (34.3–42.3)	27.9 (25.3–30.5)	<0.01
	Sport Bars	22.2 (20.5–23.9)	21.7 (19.8–23.6)	34.5 (30.6–38.4)	26.0 (23.4–28.6)	<0.01
	Sport Gels	2.4 (1.8–3.0)	4.2 (3.3–5.1)	5.9 (4.0–7.8)	3.4 (2.3–4.5)	<0.01
	Sport Bars/Gels^a^	23.1 (21.4–24.8)	23.4 (21.4–25.4)	35.5 (31.6–39.4)	27.0 (24.4–29.6)	<0.01

Abbreviations: SRNS, sport-related nutritional supplement; 95% CI = 95% confidence interval.

^a^This category was included to allow comparisons with the literature because many military studies combine sport bars and gels in reporting prevalences.

### 3.3. Adverse event reporting

Table 5 shows the prevalence of AEs reported by SMs in the BL and FU phases. The overall proportion of SRNS users reporting ≥1 AEs was 1.9 ± 0.3% in both phases. In descending order, prevalence of overall AEs was highest for sport gels, sport bars, and sport drinks in both phases.

**Table 5.** Prevalence of adverse effects reported in baseline and follow-up phases.

Adverse Effect	Any SRNS Users % ± SE (n)		Sport Drink Users % ± SE (n)		Sport Bars Users % ± SE (n)		Sport Gel Users % ± SE (n)	
	BL (*n* = 2,673)	FU (*n* = 2,350)	BL (*n* = 1,775)	FU (*n* = 1,626)	BL (*n* = 1,725)	FU (*n* = 1,386)	BL (*n* = 211)	FU (*n* = 204)
Any Adverse Effect	1.9 ± 0.3 (50)	1.9 ± 0.3 (44)	1.0 ± 0.2 (18)	1.3 ± 0.3 (21)	1.7 ± 0.3 (29)	1.4 ± 0.3 (19)	3.3 ± 1.2 (7)	2.5 ± 1.1 (5)
Palpitations	0.1 ± 0.0 (2)	0.3 ± 0.1 (8)	0.1 ± 0.1 (2)	0.4 ± 0.2 (7)	0.0 ± 0.0 (0)	0.1 ± 0.1 (2)	0.0 ± 0.0 (0)	0.0 ± 0.0 (0)
Abdominal Pain	0.4 ± 0.1 (12)	0.5 ± 0.1 (11)	0.2 ± 0.1 (4)	0.1 ± 0.1 (2)	0.4 ± 0.2 (7)	0.4 ± 0.2 (6)	0.5 ± 0.5 (1)	1.5 ± 0.9 (3)
Nausea, Vomiting	0.1 ± 0.0 (3)	0.0 ± 0.0 (1)	0.1 ± 0.1 (1)	0.0 ± 0.0 (0)	0.1 ± 0.1 (2)	0.0 ± 0.0 (0)	0.5 ± 0.5 (1)	0.5 ± 0.5 (1)
Diarrhea	0.4 ± 0.1 (11)	0.4 ± 0.1 (10)	0.1 ± 0.1 (1)	0.1 ± 0.1 (2)	0.5 ± 0.2 (8)	0.5 ± 0.2 (7)	0.5 ± 0.5 (1)	1.0 ± 0.7 (2)
Muscle Cramps, Weakness	0.2 ± 0.1 (4)	0.2 ± 0.1 (5)	0.1 ± 0.1 (1)	0.1 ± 0.1 (2)	0.1 ± 0.1 (2)	0.2 ± 0.1 (3)	0.5 ± 0.5 (1)	0.0 ± 0.0 (0)
Sleeping Problems, Insomnia	0.2 ± 0.1 (6)	0.3 ± 0.1 (6)	0.2 ± 0.1 (4)	0.2 ± 0.1 (4)	0.1 ± 0.1 (1)	0.1 ± 0.1 (2)	0.5 ± 0.5 (1)	0.0 ± 0.0 (0)
Dizzy, Confused, Lightheaded	0.0 ± 0.0 (0)	0.0 ± 0.0 (0)	0.0 ± 0.0 (0)	0.0 ± 0.0 (0)	0.0 ± 0.0 (0)	0.0 ± 0.0 (0)	0.0 ± 0.0 (0)	0.0 ± 0.0 (0)
Tingling, Numbness	0.1 ± 0.0 (2)	0.1 ± 0.0 (2)	0.1 ± 0.1 (1)	0.1 ± 0.1 (2)	0.1 ± 0.1 (1)	0.0 ± 0.0 (0)	0.0 ± 0.0 (0)	0.0 ± 0.0 (0)
Seizure, Convulsion, Tremor	0.0 ± 0.0 (0)	0.0 ± 0.0 (0)	0.0 ± 0.0 (0)	0.0 ± 0.0 (0)	0.0 ± 0.0 (0)	0.0 ± 0.0 (0)	0.0 ± 0.0 (0)	0.0 ± 0.0 (0)
Other	0.7 ± 0.2 (20)	0.5 ± 0.1 (12)	0.3 ± 0.1 (6)	0.5 ± 0.2 (8)	0.7 ± 0.2 (12)	0.4 ± 0.2 (5)	1.4 ± 0.8 (3)	0.0 ± 0.0 (0)

Abbreviations: BL,baseline phase; FU, follow-up phase; SRNS, sport-related nutritional supplement; SE, standard error.

Table 6 shows the changes in AE reporting in the FU phase. Only 11% to 17% of those reporting AEs in the BL phase also reported AEs in the FU. There were 18 BL sport drink users who reported BL AEs and of these, 8 (44%) reported continuing sport drink use in the FU phase. Of the 29 BL sport bar users who reported BL AEs, 14 (48%) reported continued sport bar use in the FU. Of the 7 BL sport gel users who reported BL AEs, 3 (43%) reported continued sport gel use in the FU.

**Table 6.** Changes in adverse event reporting at follow-up.

SRNS Category	Users Reporting AEs at Baseline				Users Not Reporting AEs at Baseline				McNemar Test *p*-Value
	SMs Reporting AE at BL and FU (Consistent Reporters)		SMs Reporting AEs at BL but not FU (Discontinued Reporters)		SMs Not Reporting AEs at BL, but Reporting AEs at FU (New Reporters)		SMs not Reporting AEs at BL or FU (Never Reporters)		
	*n*	Prevalence (%)	*n*	Prevalence (%)	*n*	Prevalence (%)	*n*	Prevalence (%)	
Any SRNS	7	14.0	43	86.0	37	1.6	2,263	98.4	0.58
Sport Drinks	2	11.1	16	88.9	19	1.2	1,589	98.8	0.74
Sport Bars	5	17.2	24	82.8	14	1.0	1,343	99.0	0.07
Sport Gels	0	0.00	7	100.0	5	2.5	192	97.5	0.39

Abbreviations: AE, adverse effect; BL, baseline phase, FU, follow-up phase; SM, service member; SRNS, sport-related nutritional supplement.

Table 7 examines SRNS use at FU by AE reporting at BL. The proportion of SMs reporting SRNS use in the FU was similar among those reported and did not report AEs at BL for all SRNS categories.

**Table 7.** SRNS use at FU by AE reporting at BL.

SRNS Category (*n*)	Reported AE at BL	*n*	Prevalence of SRNS Use at FU (% ± SE)	Odds Ratio (95% CI)	*p*-Value
Any SRNS Users at BL (*n* = 2,673)	Yes	50	62.1 ± 6.9	0.97 (0.55–1.73)	0.92
	No	2,622	64.0 ± 0.9		
Sport Drink User at BL (*n* = 1,775)	Yes	18	44.4 ± 11.7	0.67 (0.26–1.70)	0.39
	No	1,757	54.5 ± 1.2		
Sport Bar User at BL (*n* = 1,725)	Yes	29	49.9 ± 9.3	0.94 (0.45–1.95)	0.86
	No	1,696	48.3 ± 1.2		
Sport Gel Users at BL (*n* = 211)	Yes	7	42.9 ± 18.8	1.41 (0.31–6.45)	0.66
	No	204	34.8 ± 3.3		

Abbreviations: 95% CI, 95% confidence interval; AE, adverse effect; BL, baseline phase; FU, follow-up phase; SE, standard error; SRNS, sport-related nutritional supplement.

## 4. Discussion

It is important to estimate the prevalence and safety of SRNSs because of projections [9,10] that sales and therefore the number of users will increase over time. This study examined a single cohort of military SMs at two periods separated by an average of 1.3 years and found that there was a slight decline in the prevalence of SRNS use over the period. However, this decline was relatively small, amounting to <6% overall. More importantly, the distribution of individual users and non-users changed from the BL to FU phase; some SMs reported discontinuing use while others started using SRNS, thus largely maintaining prevalence in the FU period. For example, sport drink prevalence in the BL and FU phases was 31% and 28%, respectively; however, only 54% of BL users reported sport drink use in both study phases, while 46% of BL users no longer reported use in the FU. Prevalence of SRNSs was largely similar in the two phases because of “new” users in the FU. AE reporting was very low for all three categories of SRNSs amounting to only 1.9% of the users overall in both phases. Very few users who reported AEs in the BL phase also reported them in the FU phase. The similar AE prevalence in both phases was largely due to SMs who had not reported BL AEs but reported them in the FU. Slightly less than half (43–48%) of users experiencing BL AEs reported continued SRNS use in the FU. Finally, SRNS use prevalence in the FU was similar among those who did and did not report AEs at BL for all SRNS categories suggesting AEs did not deter continued usage in the FU period.

### 4.1. SRNS use prevalence

The small decline in SRNS use in the FU phase may be related to the decline in reported physical activity during the FU period. As noted above, the FU phase was conducted just after the World Health Organization declared the global COVID-19 pandemic [15]. The COVID-19 lockdowns negatively affected the US military since unit physical training was suspended, fitness facilities temporarily closed, and physical fitness testing deferred [19–21]. In the current study, the decline in reported physical activity was greater for resistance training activities than for aerobic training. Resistance training often requires specialized equipment (e.g. weight-lifting apparatuses) located in gymnasiums which were largely closed during the pandemic, while aerobic activity can be conducted with minimal personal equipment (e.g. running shoes).

We previously reported SRNS use prevalence in a much larger BL cohort (*n* = 26,681) [7] that included SMs in the current study (*n* = 5,774). The overall prevalence of SRNS use in the FU cohort (*n* = 5,774) was almost identical to that of the larger BL cohort (*n* = 26,681), 45% compared to 46%. Prevalences were also similar in the BL phase by SRNS category, 32% vs 31% for sport drinks, 27% vs 30% for sport bars, and 3% vs 4% for sport gels, respectively. Thus, participants in the current study appear very similar to the larger cohort in terms of SRNS use prevalence.

Previous studies reporting SRNS use in military and civilian populations are shown in Table 8. These data indicate that the prevalence of sport drink use ≥1 time/week among US Army personnel [4–7,11] was lower than that of Australian [22] and British [23] Army personnel. However, US Marines had the highest use prevalence in previous studies [6,7] and in the current one. Data from civilian studies where consumption ≥1time/week were quired were highly variable with use prevalence ranging from 13% to 31% [24–26]. When adult civilians were asked about sport drink “use on a regular and consistent basis,” use prevalence was 15% [27].

**Table 8.** Military and civilian studies examining sports-related nutritional supplement use prevalence.

Group	Study	Data Collection	Year Data Collected	Survey Period	Sample	Category of SRNS	Prevalence (%)
Military	Lieberman et al. 2010 [4]	Convivence sample from 11 US military posts	2006–2007	Use ≥1 time/week in last 6 months	990 US Army Personnel	Sport Drink Sport Bar or Gel	23 6
	Austin et al. 2016 [5]	Convivence sample from 8 US military posts	2010–2011	Use ≥1 time/week in last 6 months	1,750 US Air Force Personnel	Sport Drink Sport Bar or Gel	24 9
	Knapik 2016 [6]	Random sample of Navy and Marine Corps personnel	2011–2012	Use ≥1 time/week in last 6 months	700 US Navy Personnel	Any SRNS Sport Drink Sport Bar or Gel	48 36 23
					983 US Marine Corps Personnel	Any SRNS Sport Drink Sport Bar or Gel	57 51 22
	Knapik et al. 2021 [7]	US Military Dietary Supplement Use Study, stratified random sample	2018–2019	Use ≥1 time/week in last 6 months	9,789 US Air Force Personnel	Any SNRS Sport Drink Sport Bar Sport Gel Sport Bar or Gel	41 29 25 3 25
					7,935 US Army Personnel	Any SNRS Sport Drink Sport Bar Sport Gel Sport Bar or Gel	45 34 26 4 27
					3,194 US Marine Corps Personnel	Any SNRS Sport Drink Sport Bar Sport Gel Sport Bar or Gel	54 43 34 5 35
					5,763 US Navy Personnel	Any SNRS Sport Drink Sport Bar Sport Gel Sport Bar or Gel	44 30 28 4 29
	Austin et al. 2015 [11]	Convivence sample from US 13 locations	2010–2011	Use ≥1 time/week in last 6 months	1,033 US Coast Guard Personnel	Sport Drink Sport Bar or Gel	25 12
	Kullen et al. 2019 [22]	Convivence sample of Australian Army personnel	Not Reported	Use ≥1 time/week	667 Australian Army Personnel	Sport Drink Sport Bar Sport Gel	42 6 3
	Casey et al. 2014 [23]	Convivence sample from 11 UK military locations	2010–2011	Current use	3,168 UK Army personnel	Sport Drink Sport Bar Sport Gel	49 18 10
Civilian	Larson et al. 2015 [24]	Initial sample from 31 schools in Minneapolis/St Paul; participants followed 10 years later	2008–2009	Consumption ≥1 time/week	2,287 young adults, aged 20–31 years	Sport Drink	31
	Cordrey et al. 2018 [25]	National Youth Physical Activity and Nutrition Survey (NYPANS) and Youth Risk Behavior Survey (YRBS)	2010	Consumption ≥1 time/week in last 7 days	NYPANS − 4,529 High School Students ≥17 years	Sport Drink	15
					YRBS − 4,583 High School Students ≥17 years	Sport Drink	13
	Zytnick et al. 2015 [26]	Summer Consumer Styles Survey	2011	Consumed ≥1 time/week in past 7 days	3,929 adults, aged 18 to >65 years	Sport Drink	22
	Costello et al. 2015 [27]	Natural Marketing Institute Supplement/Over-the-Counter/Prescription Survey	2011	Use on a regular and consistent basis	3,255 adults, aged 18 to >65 years	Sport Drink Sport Bar	15 11

Abbreviations: SRNS, sport-related nutritional supplement; US, United States; UK, United Kingdom.

The prevalence of sport bar use among US Army personnel [7] was higher than among British [23] and Australian [22] Army personnel and much higher than among American civilians [27]. Among the US military services, Marines had the highest sport bar use prevalence in our previous BL study [7] and in the current FU. Sport gel use prevalence was highest among British soldiers [23] and similar among Australian [22] and US [7] soldiers. It should be noted that US Army [4], Air Force [5], and Navy/Marine Corps [6] data were collected in 2006–2007, 2010–2011, and 2011–2012, respectively. Comparing these data with those collected in 2018–2019 [7] suggests that sport drink use has increased over time in the Air Force and Army and decreased slightly in the Marine Corps and Navy. Use of sport bars and gels appears to have increased in all services, especially the Air Force and Army.

A meta-analysis [3] of 17 studies encompassing a wide range of athletic samples found that 28% (95% CI = 18–24%) and 34% (95% CI = 22–47%) of athletes reported using sport drinks and sport bars, respectively. However, as indicated by the 95% CIs, there was a wide range of prevalences depending on the sport, country of origin, and the year the data were collected. The prevalence of sport drink use in the present study (all US services combined) was similar to that of this broad athletic sample [3], but use of sport bars was somewhat lower.

As noted above, use of SRNS was highest among Marine Corps participants in the current study. Marine Corps participants were younger, performed more resistance training, and were more likely to be men, compared to the other services (data not shown). Our previous study [7] indicated that these factors increased the odds of SRNS use.

### 4.2. Adverse effects

We previously reported [7] that the prevalence of AEs in our large BL sample (*n* = 26,681) was low, 1.3%, 1.6%, and 2.8% among consumers of sport drinks, sport bars, and sport gels, respectively. In a previous study of Navy and Marine Corps personnel conducted by our group [6], 3.7% and 3.1% of the consumers reported ≥1 AE for sport drinks and for sport bars/gels, respectively. In the current study, AEs were similarly low in both BL and FU phases, but very few consumers reported AEs in both phases for any of the SRNSs. For example, among sport drink users only 2 SMs reported AEs in both phases while 19 participants who reported AEs in the FU phase did not report any AEs in the BL. Further, slightly less than half (43–48%) of individuals who experienced BL AEs reported continued use of SRNS in the FU and FU usage prevalence was similar among those who did and did not use experience BL AEs. These data suggest that AEs may have been transient (i.e. experienced in only one study phase) or mild and did not deter many users from continuing consumption.

The relatively low AE prevalence for SRNSs contrasts with dietary supplements for which 8% to 29% of military SMs have previously reported AEs [6,12,28–34]. We earlier speculated [7] that the lower AE prevalence for SRNS may be related to the authority the US Food and Drug Administration (FDA) has to control these two classes of supplements. Nutritional supplements like SRNSs are regulated by the FDA as foods under Federal Food, Drug, and Cosmetic Act of 1938, as well as the amendments to that act since its initiation [35,36]. Under this act, a food is considered “safe” if it is judged as such by competent scientists under its intended conditions of use [37]. In contrast, dietary supplements are regulated under the Dietary Supplement Health and Education Act of 1994 [38] which limits the authority of the FDA. Manufacturers must notify the FDA before marketing a new dietary supplement, but FDA approval is not required for retailing the product. The FDA must demonstrate a specific product is unsafe, although manufacturers are required to notify the FDA about serious AEs [39,40]. Regulation of SRNS as foods appears to result in a lower incidence of AEs than DSs which are controlled in a much more limited manner.

### 4.3. Strengths and limitations

This is the first longitudinal study following SRNS use in the same cohort over time. The sample size was relatively large and the SRNS prevalence values similar to that of larger sample (*n* = 26,681) [7] suggesting the current sample (*n* = 5,776) was representative in terms of SRNS use prevalence. The questionnaire used in both phases was identical and based on previous questionnaires designed for obtaining similar supplement data from SMs [16]. Nonetheless, there were some limitations. All data were self-reported and suffer the usual weaknesses associated with this method including recall bias, social-desirability bias, errors in self-observation, and inadequate recall [41,42]. An attempt was made to mitigate some of these factors by requesting SRNS use only in the last 6 months. In self-reporting AEs, participants were limited to nine specific AE categories, although they could report and describe other AEs that were subsequently categorized by the investigators.

## 5. Conclusions

Overall SRNS use prevalence decreased slightly in the FU period and this could have been at least partly associated with reductions in physical training and COVID-19 lockdowns that suspended military unit physical training, closed fitness facilities, and postponed physical fitness testing. A large proportion of SMs changing their use patterns in the FU phase with some discontinuing use and others initiating use. The AE incidence was very low, amounting to 1.9% of the users in both BL and FU phases. Very few SMs reported AEs in both phases and many users who reported BL AEs continued using SRNSs in the FU suggesting AEs were transitory. The more stringent FDA regulation of these sport supplement products may account, at least in part, for the low prevalence of reported AEs compared to dietary supplements.

## Data Availability

The datasets generated and/or analyzed during the current study are not publicly available due to US government restrictions but are available from the corresponding author on reasonable request.

## List of Abbreviations

95% CI: 95% confidence interval
AE: adverse effect
BL: baseline
BMI: body mass index
COVID-19: coronavirus disease 2019
FDA: Food and Drug Administration
FU: follow-up
SM: service member
SE: standard error
SRNS: sports-related nutritional supplement
US: United States
